# An ELISA DYRK1A non-radioactive kinase assay suitable for the characterization of inhibitors

**DOI:** 10.12688/f1000research.10582.2

**Published:** 2017-03-24

**Authors:** Yong Liu, Tatyana Adayev, Yu-Wen Hwang

**Affiliations:** 1 Molecular Biology Department, New York State Institute for Basic Research in Developmental Disabilities, Staten Island, NY, USA

**Keywords:** non-radioactive kinase assay, EGCG, harmine, inhibitor screening

## Abstract

The DYRK1A (dual specificity tyrosine phosphorylation-regulated kinase 1A) gene encodes a proline-directed Ser/Thr kinase. Elevated expression and/or altered distribution of the kinase have been implicated in the neurological impairments associated with Down syndrome (DS) and Alzheimer’s disease (AD). Consequently, DYRK1A inhibition has been of significant interest as a potential strategy for therapeutic intervention of DS and AD. Many classes of novel inhibitors have been described in the past decade. Although non-radioactive methods for analyzing DYRK1A inhibition have been developed, methods employing radioactive tracers are still commonly used for quantitative characterization of DYRK1A inhibitors. Here, we present a non-radioactive ELISA assay based on the detection of DYRK1A-phosphorylated dynamin 1a fragment using a phosphorylation site-specific antibody. The assay was verified by the use of two well-characterized DYRK1A inhibitors, epigallocatechin gallate (EGCG) and harmine. The IC _50_s for EGCG and harmine determined by the ELISA method were found to be comparable to those previously measured by radioactive tracing methods.  Furthermore, we determined the mode of inhibition for EGCG and harmine by a modification of the ELISA assay. This assay confirms the mode of inhibition of EGCG (non-ATP-competitive) and harmine (ATP-competitive), as previously determined. We conclude that the ELISA platform demonstrated here is a viable alternative to the traditional radioactive tracer assays for analyzing DYRK1A inhibitors.

## Introduction

The human DYRK1A gene ^1^ is mapped to a region of chromosome 21 implicated in Down syndrome (DS) ^2^. DS, the most common chromosomal abnormality associated with birth defects and developmental disabilities, is caused by full or partial trisomy of chromosome 21 ^3^. Almost all DS cases inevitably lead to the development of Alzheimer’s disease (AD)-type pathology ^4^. Transgenic mice carrying an extra copy of DYRK1A have been shown to exhibit symptoms similar to DS, including brain abnormalities, neurodevelopmental delay, and memory impairments ^5– 7^. DYRK1A in the brain displays a distinct structure-specific distribution pattern ^8^, and it interacts with an array of factors involved in neuronal development, proliferation, and differentiation ^9^. The level of DYRK1A is elevated in a gene dosage-dependent manner in DS, suggesting that the protein not only plays an important role in regulating normal brain functions, but also in the etiology of DS ^8, 10^.

DYRK1A has been linked to neurofibrillary degeneration and β-amyloidosis of AD ^11^. DYRK1A was shown to phosphorylate microtubule-associated protein tau at T212 to prime tau for subsequent phosphorylation by GSK-3β at S308 ^12, 13^. This inhibits tau’s ability to stimulate microtubule assembly and promotes self-aggregation, like abnormally hyperphosphorylated tau in AD brain ^13^. DYRK1A was also found to phosphorylate amyloid precursor protein (APP) at T668 ^14^ and presenilin-1 at T354 ^15^, which correlated with increased cleavage of APP by β and γ secretases ^15, 16^ respectively, and leads to the formation of neurotoxic β-amyloid peptides (Aβ). Moreover, Aβ is shown to be involved in a positive feedback loop for promoting DYRK1A expression, which may further accelerate production of Aβ ^17^.

The collected evidence suggests that DYRK1A is a potential drug target for the treatment of DS and AD. To this end, many classes of DYRK1A inhibitors, both natural and synthetic, have been tested ^18– 20^. The potency of such inhibitors has mostly been analyzed using radioactive tracer methods despite the availability of non-radioactive assays ^21, 22^. It may be that these methods typically require multiple steps, which is undesirable for screening. Here, we describe a simple ELISA assay for DYRK1A inhibition using dynamin 1a and its phosphorylation site antibody for detection ^23, 24^. The ELISA assay has been verified using two known DYRK1A inhibitors and found to be consistent with radioactive tracer methods.

## Methods

### Materials

Epigallocatechin gallate (EGCG; #70935) and harmine (#10010324) were obtained from Cayman Chemical. Para-nitrophenyl phosphate (PNPP) tablets and diethanolamine substrate buffer were purchased from Thermo-Fisher Scientific. EGCG and harmine were initially prepared as 50 mM stock in 100% DMSO. Working solutions of EGCG and harmine (0.01 μM – 3.2 μM) were prepared from stocks in 2% DMSO by serial dilution. Dynamin 1a pS857-specific mouse mAb 3D3 (RRID: AB_2631263, the antibody has been deposited with the Developmental Studies of Hybridoma Bank) was prepared as described ^24^. The antibody was partially purified from ascites using Bakerbond ABx resins (#7269-02) before use. Anti-dynamin mAb Hudy-1 (RRID: AB_309677) ^25^ was obtained from EMD Millipore. Alkaline phosphatase (AP) conjugated goat anti-mouse IgG secondary antibody (#115-055-146) was purchased from Jackson ImmunoResearch Laboratories, Inc.

### Kinase and substrate preparation

6xHis tagged rat truncated DYRK1A containing residues 1-497 (HT-497) was used for all assays. This truncation preserves activity of DYRK1A ^26, 27^. A bacterial HT-497 expression vector was constructed as follows. The truncated DYRK1A gene was first obtained by PCR from the GST-DYRK1A vector ^23^ using a pair of primers for producing the DYRK1A fragment with a Cla I site plus a 6XHis tag (cctatcgatgcatcatcatcatcatcaccatacaggaggagagacttc) at the start codon and an in-frame termination at codon 498 plus a Xho I site (ggactcgagtcaagggctggtggacacactgtt), respectively, at 5’ and 3’ ends. PCR was performed in 50 μl mixture containing 10 ng template, 0.2 μg of each primer, 0.2 mM ATP, and PfuUltra (Agilent Technologies), as recommended by the supplier. Amplification was conducted with 20 cycles of the following steps: 94°C, 30-sec, 72°C 90-sec, and 62°C 30-sec. Custom primers were purchased from Integrated DNA Technologies. The resulting amplicon was then cloned into a modified T7 promoter-driven vector pND1 ^28^ via the Cla I and Xho I sites, as described ^29^. Proline rich domain (PRD, residues 746–864) of dynamin 1a was also prepared as N-terminal tagged 6xHis fusion protein (HT-PRD) exactly as described above. The PRD fragment was first produced from the semi-synthetic dynamin 1a gene ^24^ by PCR using a pair of primers, (aggatcgatgcatcatcatcatcatcataacacgaccaccgtcagcacg) and (aggctcgagtcataggtcaaaaggtggtcg) for subsequent cloning into expression vector pND1, like HT-497. It should be noted that the HT-PRD construct carries an inadvertent F862L substitution. Both pHT-497 and pHT-PRD have been deposited with Addgene.

Both HT-497 and HT-PRD were expressed and purified using TALON metal affinity resin (Clontech Laboratories) under native conditions as described ^29^. Proteins were quantified by Bradford method ^30^ and stored at -80°C until use.

### ELISA assay

**Figure 1.**
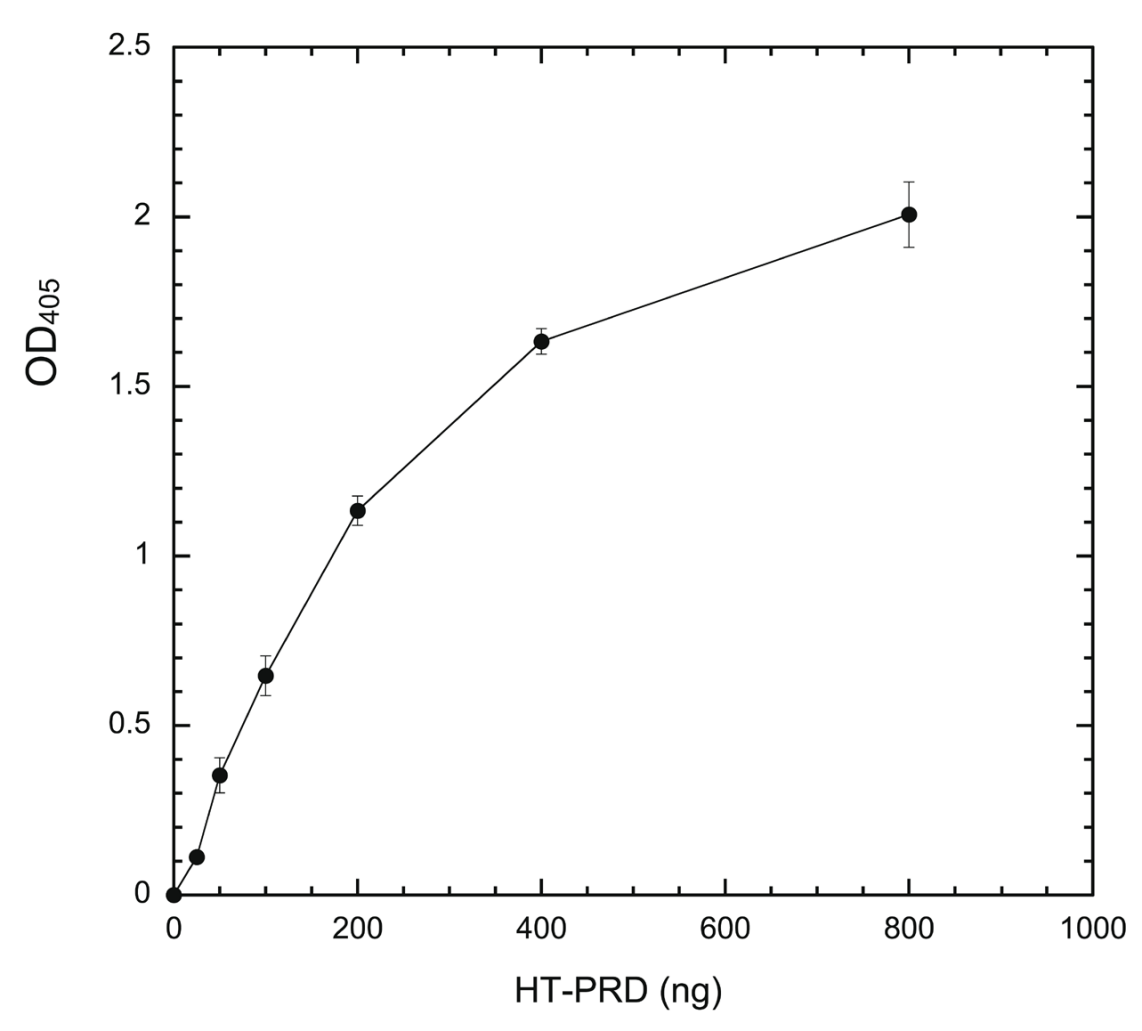
Coating ELISA plate with HT-PRD. Wells were incubated with indicated amounts of HT-PRD (0, 25, 50, 100, 200, 400, and 800 ng/well) at 4°C overnight and the level of coated proteins was then detected with anti-dynamin mAb Hudy-1 by following the sandwich ELISA protocol, as described in Methods (n = 4 for each data point).

**Figure 2.**
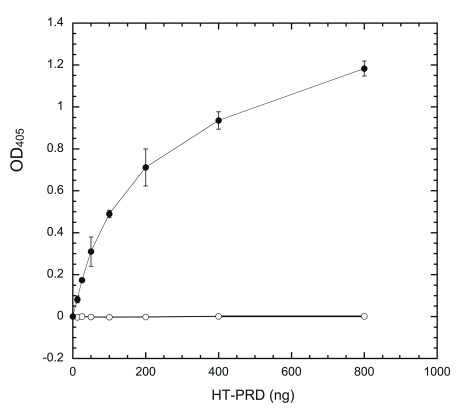
Phosphorylation of coated HT-PRD by DYRK1A. Wells were coated with indicated amounts of HT-PRD (0, 25, 50, 100, 200, 400, and 800 ng/well) and then subjected to extensive DYRK1A phosphorylation *in situ* by incubation with 80 ng of HT-497 at 30°C for 60 min. The level of S857 phosphorylation was then detected with 3D3 following the sandwich ELISA protocol, as described in Methods (n = 4 for each data point). Filled circles (●), with kinase; empty circles (○), without kinase.

Substrate, HT-PRD, was diluted in dilution buffer (25 mM Tris-HCl, pH 7.4 and 100 mM NaCl) to a concentration of 2 ng/μl (or higher as in Figure 1 and Figure 2) and used to coat a 96-well plate (BD Falcon #353072) with 100 μl per well (200 ng/well unless otherwise indicated) at 4°C overnight. Unbound materials were washed away with dilution buffer and wells were blocked with 150 μl blocking buffer (2% BSA, 1X PBS, and 0.25% Tween 20) at room temperature for 60 min. After blocking, wells were washed extensively with dilution buffer before subjecting to phosphorylation. DYRK1A phosphorylation was performed in wells with 100 μl reaction mix containing 25 mM HEPES, pH7.4, 100 mM NaCl, 5 mM MgCl _2_, 100 μM ATP (Sigma-Aldrich Chemicals), inhibitor if needed, and 5 ng HT-497 (unless otherwise indicated). Reactions were initiated by adding HT-497 and continued for 30 min (unless otherwise indicated) at 30°C. For time course experiments, reactions were terminated by the addition of 20 mM EDTA at the indicated time points. A set of inhibition experiments typically consists of a no-inhibitor control plus a series of eight inhibitor concentrations (0.001 μM - 3.2 μM final). Each point was run in duplicate with DMSO present in all assays at 0.2% final concentration. DMSO, up to 2%, does not affect the potency of EGCG and harmine. HT-PRD phosphorylation was subsequently determined by the sandwich antibody staining protocol, first with 100 μl mAb (60 min at room temperature) then with 100 μl AP-linked anti-mouse secondary antibody (60 min at room temperature), followed by colorimetric reaction with 100 μl PNPP solution. The extent of AP reaction was monitored at λ=405 nm. For Hudy-1 staining, wells were coated, blocked, and then stained with the antibody (1:3000 dilution) for colorimetric detection as described above.

Dilution factors for both mAb 3D3 and secondary antibody were pre-determined for each batch of antibody to ensure that neither antibody was limiting in the assay. A stock to be determined was serially diluted (from 1000 to 256,000-fold) and each dilution was used together with a non-limiting concentration of the other antibody to assess the level of HT-PRD phosphorylated under standard ELISA reaction conditions without inhibitor (see Results and Discussion). OD _405_ readings were normalized to the 1000-fold dilution and plotted against the dilutions of the testing antibody. Dilutions in the normalized OD _405_ plateau can be used for the assay. We routinely use 1:3000 dilutions for ABx purified 3D3 stock (~1.5 mg/ml) and 1:2000 dilutions of commercial secondary antibody for the assay.

### Data analysis

Data transformation, calculation, plotting, curve fitting, and IC _50_ calculation were performed in KaleidaGraph ( http://www.synergy.com/wordpress_650164087; Mac version 4.1). Data was corrected for background (readings from wells with only PNPP) before subsequent manipulations. To determine IC _50_, the residual DYRK1A activity was first calculated as the ratio to the no-inhibitor control in that set. The resulting residual activity was then plotted against its corresponding inhibitor concentrations in semi-log graph and the plot was fit to the sigmoidal equation, y = a+(b-a)/(1+(x/c) ^d^), for IC _50_ calculation. Data throughout the article are presented as mean ± standard error of mean.

### ATP competition assay

The standard ELISA protocol was modified to run under conditions allowing a constant inhibitor to compete against varying ATP concentrations in inhibiting DYRK1A. Briefly, a set of competition experiments had four DYRK1A assays in the presence of different ATP concentrations (100, 200, 400, or 800 μM) with a single fixed concentration of the inhibitor to be tested. An identical set, except without inhibitor, was performed in parallel (no-inhibitor controls). The inhibitor concentration used was roughly twice the IC _50_ of the inhibitor. All other procedures of the assay are unchanged. Residual kinase activity with the inhibitor at each ATP concentration was first calculated as a percentage of the corresponding no-inhibitor control. The residual kinase activity was subsequently converted to inhibition potency as the difference from 1. The value for each ATP concentration was then normalized to the inhibition potency at 100 μM ATP and plotted.

## Results and discussion

### Development of the non-radioactive DYRK1A ELISA assay

We chose to follow the ELISA-based protocol ^31, 32^ in developing our assay, by immobilizing the substrate followed by kinase phosphorylation in the wells, as this format offers the advantage of a simple, proven design versus other non-radioactive approaches ^33^. Like many non-radioactive approaches ^33^, our assay relies on a phospho-specific antibody to differentiate between phosphorylated from un-phosphorylated substrates. The antibody used in the assay, mAb 3D3, was raised against DYRK1A-phosphorylated Dynatide 3; a peptide derived from the DYRK1A phosphorylation site of dynamin 1a at S857 ^24^. 3D3 has been shown to recognize only pS857-dynamin 1a in rat brain extracts upon extensive phosphorylation ^24^.

Dynatide 3, which is used routinely as a substrate to measure DYRK1A activity by the radioisotope/filter binding method ^27, 34, 35^, was first tested as a substrate. Unfortunately, it failed to produce any signal upon phosphorylating coated Dynatide 3 with DYRK1A, presumably due to a lack of peptide coating. To circumvent this problem, we used a 6X histidine-tagged PRD of dynamin 1a (HT-PRD) as a DYRK1A substrate. This fragment coats wells in a concentration-dependent manner and the amount of immobilized proteins, as revealed by mAb Hudy-1 staining, are proportional to input proteins up to 200 ng/well of HT-PRD (15 pmole/well) ( Figure 1).

We then examined whether immobilized HT-PRD is accessible to DYRK1A. Wells coated with varying amounts of HT-PRD were subjected to exhaustive phosphorylation *in situ* with excess DYRK1A (HT-497) (see Methods) for 60 min and probed with excess (non-limiting) mAb 3D3 and secondary antibodies (see below in Figure 5). Phosphorylated immobilized HT-PRD was recognized by 3D3. The 3D3 signal was elevated in response to increasing input of HT-PRD ( Figure 2, filled circles) initially, then leveled off, closely resembling the response of substrate coating ( Figure 1). As controls, uncoated wells phosphorylated by HT-497 ( Figure 1) and coated HT-PRD, processed without HT-497, produced no detectable signals ( Figure 2, empty circles). These results indicate that immobilized HT-PRD is phosphorylatable by DYRK1A and that the output of the assay requires DYRK1A phosphorylation.

**Figure 3.**
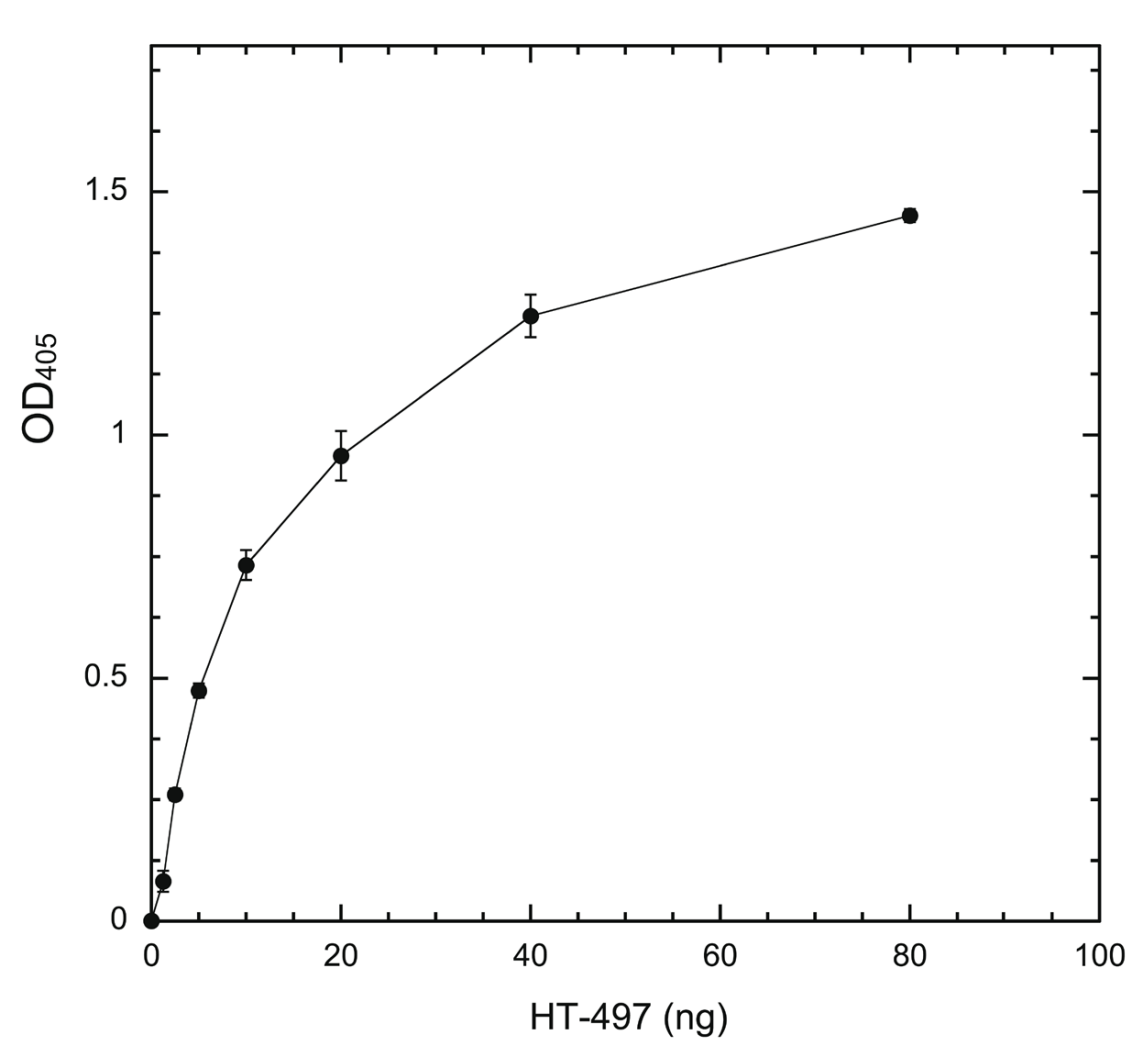
DYRK1A concentration-dependent phosphorylation of coated HT-PRD. Wells were coated with 200 ng/well HT-PRD and then subjected to DYRK1A phosphorylation with varying amounts of HT-497 (1.25, 2.5, 5, 10, 20, 40, and 80 ng) at 30°C for 30 min. The level of S857 phosphorylation was then detected with 3D3 as described in Methods (n = 6 for each data point).

**Figure 4.**
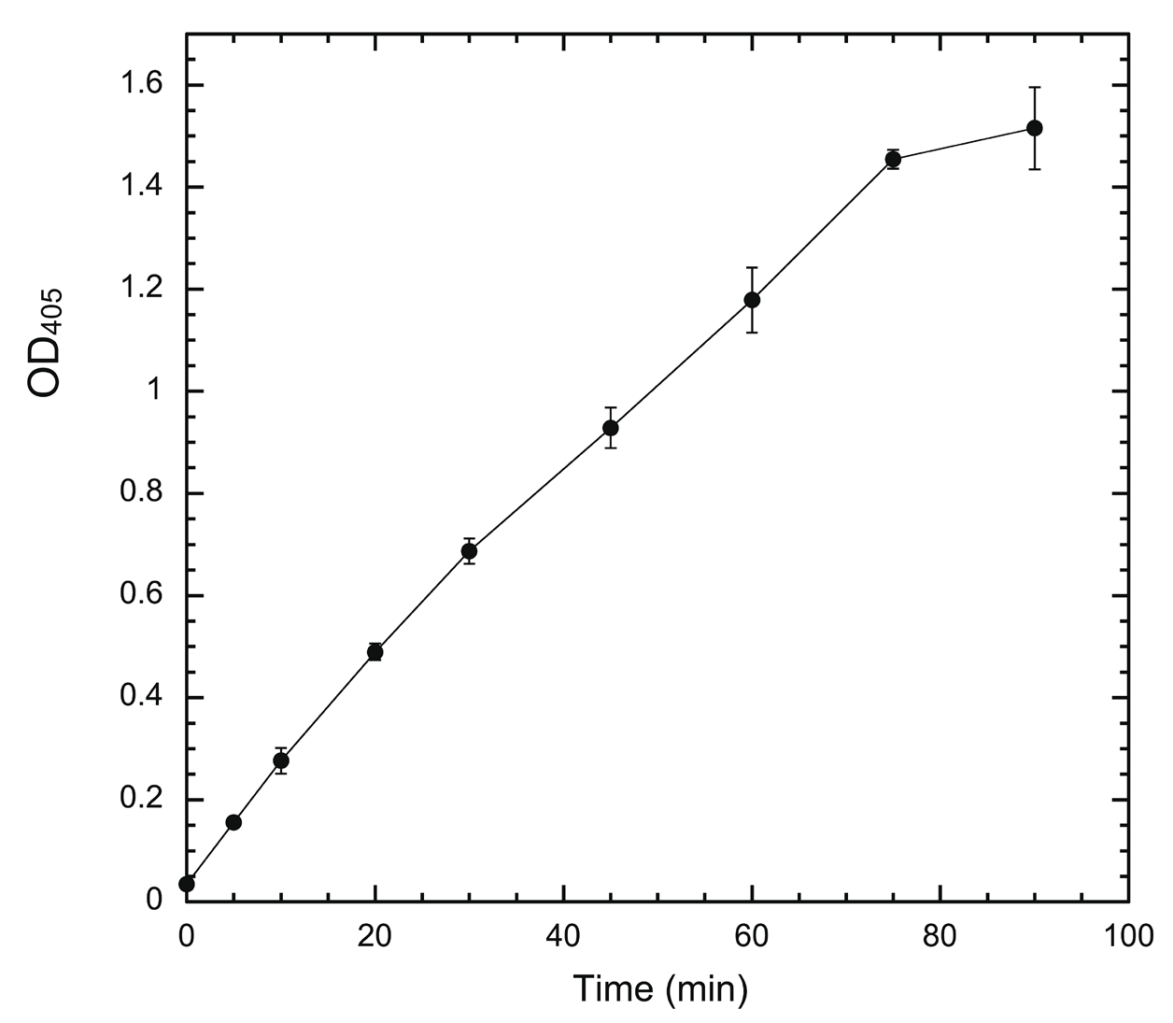
Time-course phosphorylation of coated HT-PRD by DYRK1A. Wells were coated with 200 ng/well of HT-PRD and then subjected to DYRK1A phosphorylation with 5 ng HT-497 at 30°C. The reactions were terminated at the indicated time points (0, 5, 10, 20, 30, 45, 60, 75, and 90 min) by the addition of 20 mM EDTA. The level of S857 phosphorylation was then detected with 3D3 as described (n = 3 for each data point).

If a system is to be useful in determining inhibitor potency quantitatively, the output of the system must be solely dependent on DYRK1A activity in a linear fashion. We used a fixed amount of coated HT-PRD (200 ng/well) to identify the proper conditions. The system response to changes of HT-497 was first examined ( Figure 3). Our ELISA system produces sufficient signal to be readily distinguished from the noise of no-kinase control, with ~1 ng HT-497 (~17 fmole) phosphorylation at 30°C for 30 min. The output (the equivalent of reaction rate) is elevated accordingly as enzyme concentration increased, but the ratio of elevation to enzyme concentration, in proportion to enzyme, is progressively reduced ( Figure 3). This is a typical enzyme concentration-dependent reaction profile when the substrate becomes the limiting factor ^36^. Time-course experiments were subsequently conducted with 5 ng HT-497, as the highest enzyme concentration producing a near-linear enzyme-dependent response. The output was found to be linear with reaction times up to about 75 min ( Figure 4). Therefore, we use the following standard conditions [200 ng of substrate, 5 ng HT-497 (0.82 nM), 100 μM ATP, and 30 min kinase reaction at 30°C] for all subsequent experiments. The Z’-factor for the assay performed under standard conditions was estimated and found to be greater than 0.7 ( Supplementary Table).

**Figure 5.**
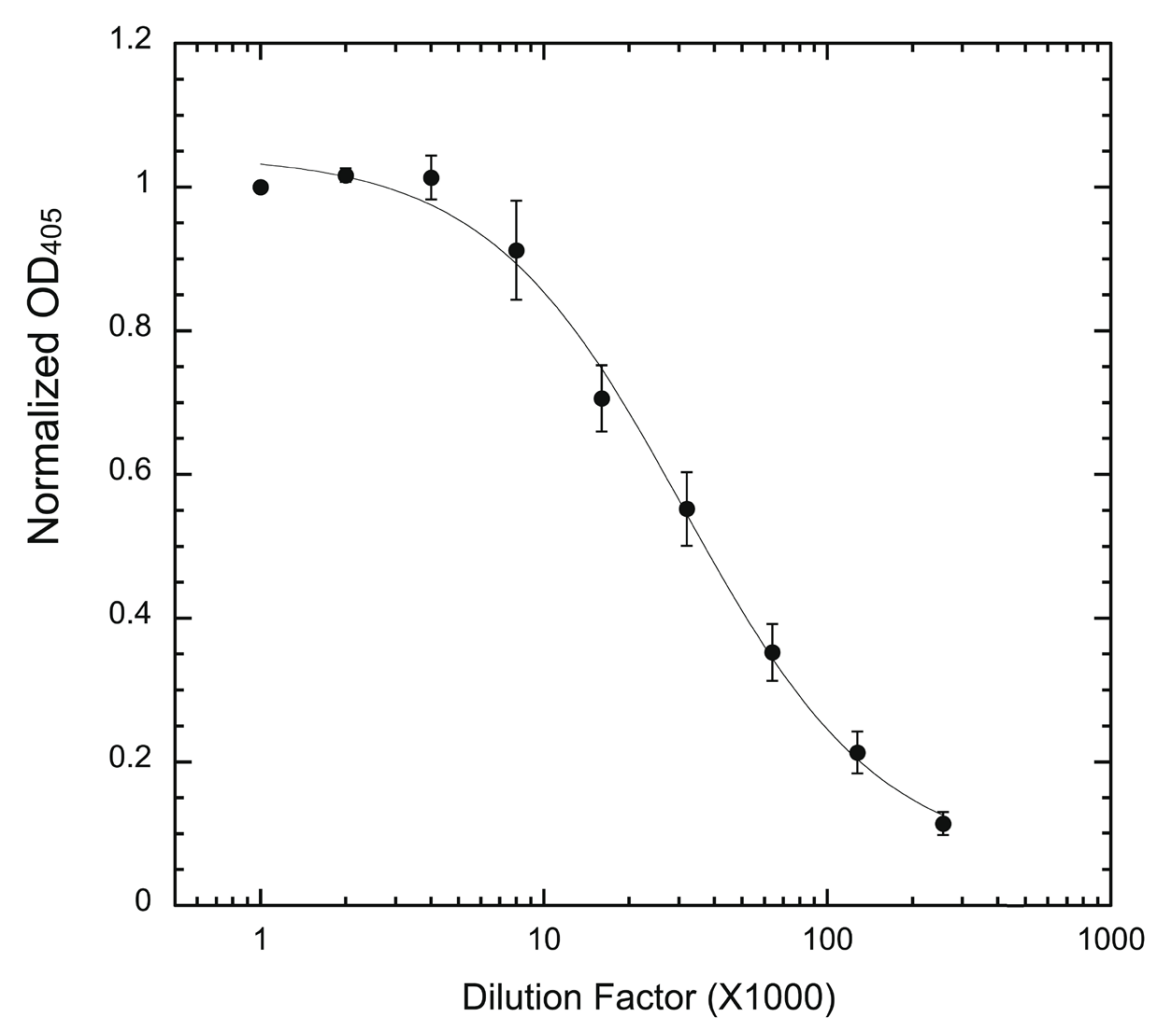
3D3 dilution factor determination. Wells were coated with 200 ng/well HT-PRD and then subjected to phosphorylation with 5 ng HT-497 under the standard reaction conditions. 3D3 to be tested was serially diluted (from 1000 to 256,000x) and used to probe the phosphorylated wells, followed by secondary antibody as described. Normalized OD _405_ was calculated (see Methods) and used for plotting (n = 9 for each data point).

To support accurate measurement of IC _50_, the amounts of antibody, both 3D3 and secondary antibody, must not be limiting. Otherwise, immunostaining will most likely under-report the actual phosphorylation level at lower concentrations of inhibitor, which could skew the IC _50_ calculation. Therefore, each batch of antibody was titered to determine the maximal dilution can be used. As shown for titering of 3D3, when the antibody is limiting (provided that a non-limiting concentration of secondary antibody is used), the readout will increase upon addition of 3D3 until a plateau indicating saturation ( Figure 5). Only dilutions that produce readout in the plateau (non-limiting) region should be used for the assay ( Figure 5). Dilution factors for the secondary antibody were similarly determined ( Supplementary Figure).

### Measuring IC _50_ and the mode of inhibition for DYRK1A inhibitors

We subsequently tested the system by examining two well-characterized inhibitors, EGCG and harmine ^27, 35^. A typical inhibition profile conducted by the ELISA method for EGCG ( Figure 6A) and harmine ( Figure 6B) follows a sigmoidal function. IC _50_s for EGCG and harmine determined by the ELISA method were 0.215 ± 0.024 μM and 0.107 ± 0.018 μM, respectively. These values are comparable to those obtained earlier by us and others with different substrates and protocols, including the radioisotope/filter binding assay, generally regarded as the gold standard for kinase inhibition assays ( Table 1) ^22, 37– 39^. The results obtained from this ELISA assay appear to be as reproducible as any given enzymatic assay. These results confirm that our ELISA platform is a valid system for quantitative characterization of DYRK1A inhibitors.

**Figure 6A.**
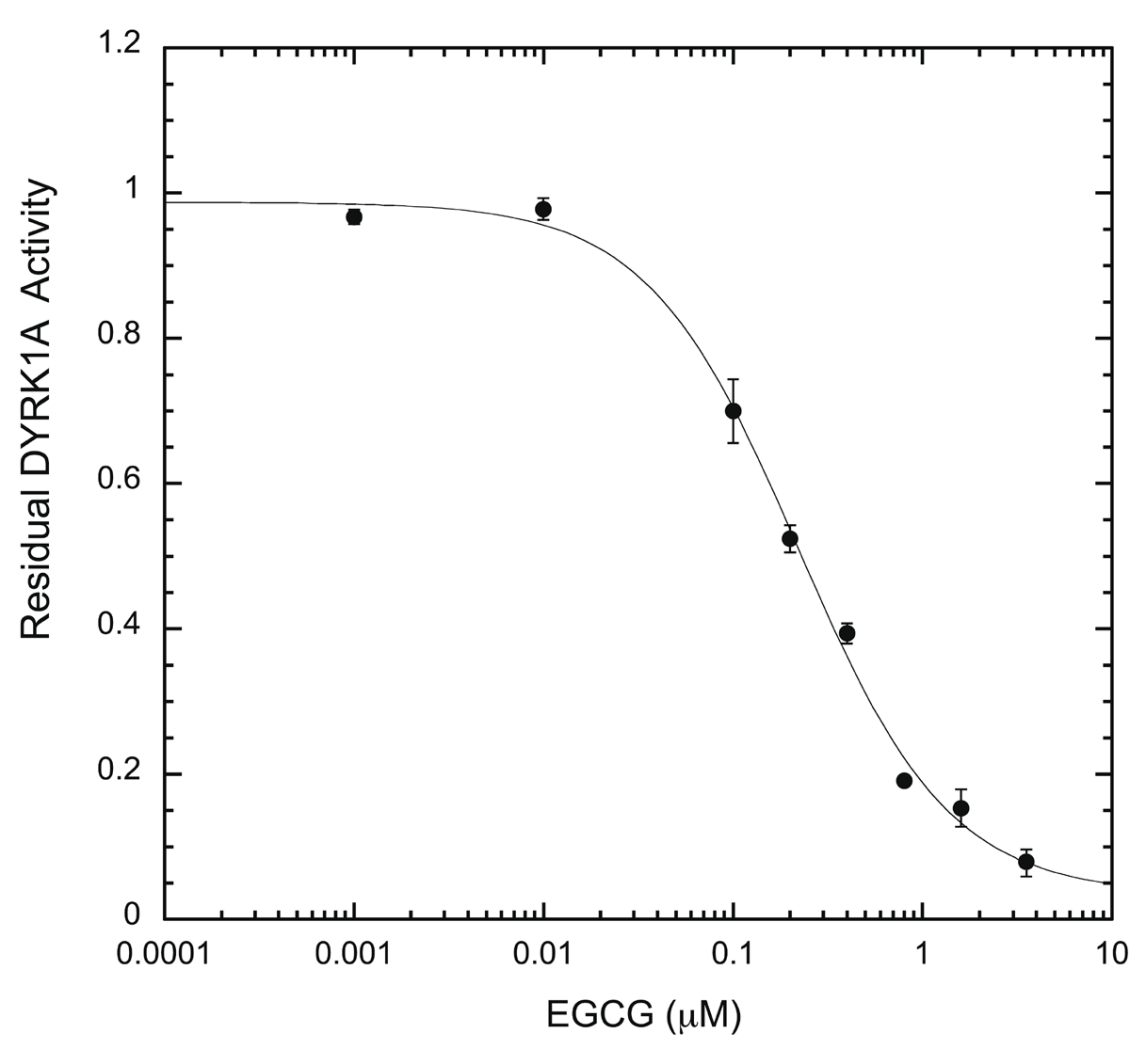
Epigallocatechin gallate (EGCG) inhibition profile. EGCG inhibition assays were performed in the presence of serially diluted EGCG (0.001 – 3.2 μM) under the standard reaction conditions. DYRK1A activity at any given EGCG concentration was calculated as a ratio to the activity of the no-inhibitor control and plotted on the Y-axis, versus EGCG concentration. IC _50_ was calculated from the plot after curve fitting, as described in Methods (n = 6 for each data point).

**Figure 6B.**
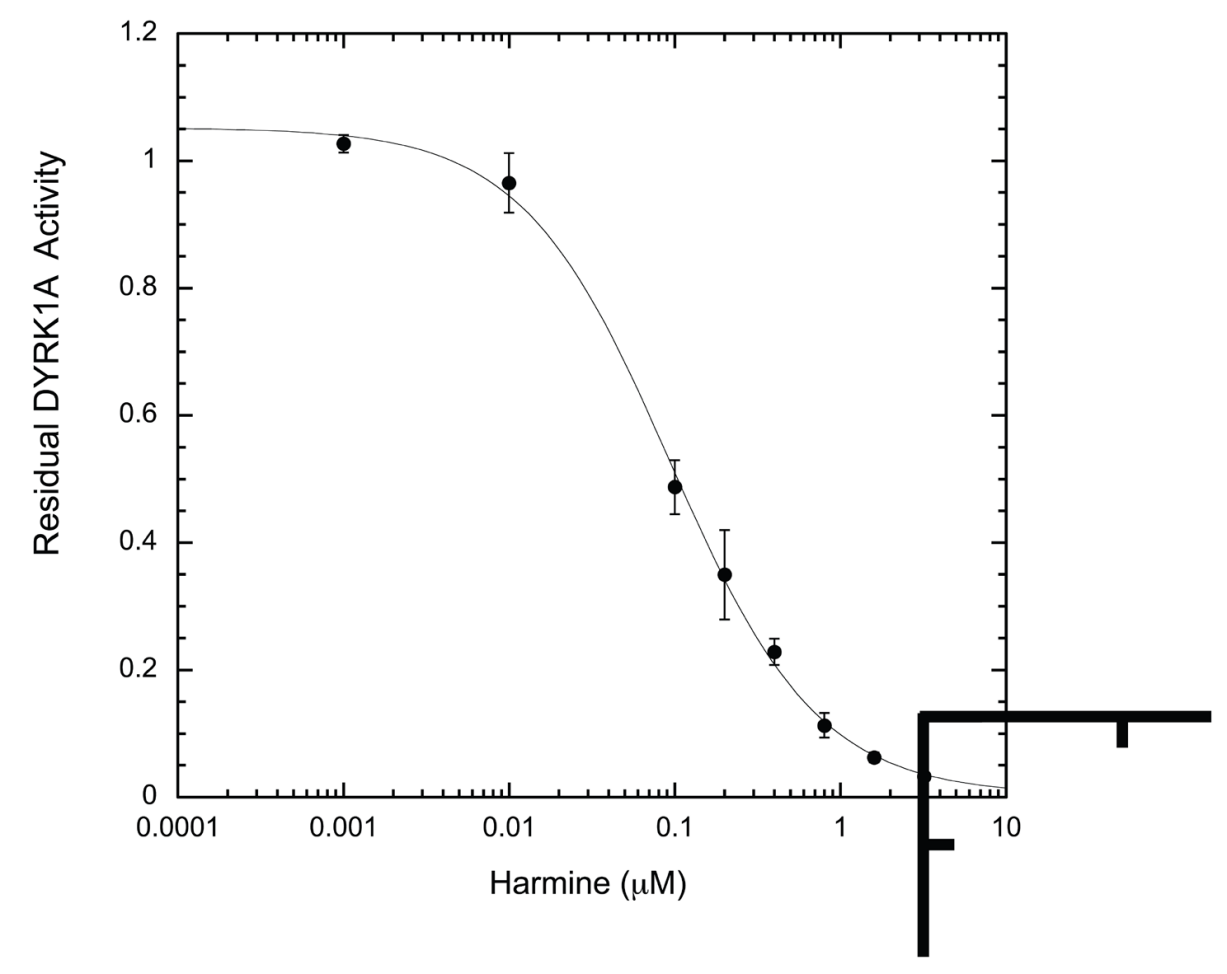
Harmine inhibition profile. Harmine inhibition was conducted and analyzed exactly as described in Figure 6A for EGCG (n = 6 for each data point).

**Table 1.** List of IC _50_ for epigallocatechin gallate (EGCG) and harmine obtained by different approaches.

Inhibitor IC _50_ (μM)	DYRK1A ^*^	Substrate	[ATP] (μM)	Detection
EGCG				
0.33	1	Woodtide ^40^	100	Radioisotope ^37^
0.31 (K _I_)	1	FAM-Woodtide	100	HPLC/Fluorescence ^22^
0.43	2	Dynatide 3 ^24^	50	Radioisotope ^27^
0.215 ^#^	3	HT-PRD	100	ELISA (this study)
Harmine				
0.08	1	Woodtide	100	Radioisotope ^38^
0.033	1	DYRKtide ^41^	100	Radioisotope ^39^
0.20 (K _I_)	1	FAM-Woodtide	50	HPLC/Fluorescence ^22^
0.075	2	Dynatide 3	50	Radioisotope ^35^
0.107 ^#^	3	HT-PRD	100	ELISA (this study)

^*^DYRK1A used for the assay:
1: GST-DYRK1A (residues 1-499) ^26^
2: GST-DYRK1A (residues 1-497) ^27^
3: HT-DYRK1A (residues 1-497) (this study)
^#^Reported IC _50_ was the average of three independent sets of duplicate assays (n = 6).

**Figure 7.**
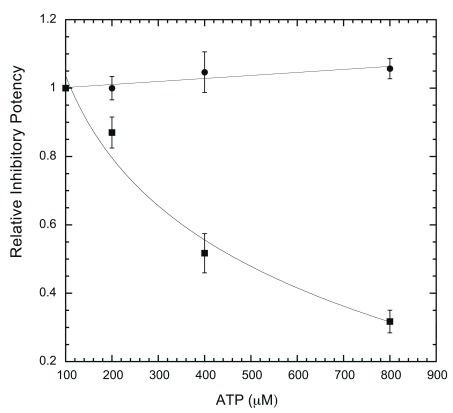
ATP competition assay. For each inhibitor (epigallocatechin gallate (EGCG) and harmine), assays were conducted with a single concentration of inhibitor in four different ATP concentrations (100, 200, 400, 800 μM) and quantified as described in Methods. The inhibitory potency at three other ATP concentrations was calculated as relative to that at 100 μM ATP and used to plot against ATP concentrations. Inhibitor concentrations used in the assays were 0.4 μM for EGCG (●) and 0.2 μM for harmine (▪) (n = 6 for each data point).

We then modified the ELISA protocol to run the assays under a single concentration of inhibitor with varying ATP concentrations, to determine whether ATP is competitive against the inhibitor in question. This allows the efficacy of inhibition to be evaluated with changing ATP. ATP is expected to influence the potency of competitive inhibitors, but not that of non-competitive inhibitors. As shown in Figure 7, harmine loses potency against DYRK1A when ATP is increased from 100 to 800 μM, indicating an ATP-competitive mode, while EGCG potency remains essentially unchanged (non-ATP-competitive). The inhibitory modes for harmine and EGCG revealed by the ELISA assay are the same as previously reported by the radioisotope/filter binding method ^27, 35^. This further validates the ELISA assay.

Raw data for Figures 1–7 and Supplementary Figure are supplied in Excel format (zipped file) Click here for additional data file. Copyright: © 2017 Liu Y et al. 2017 Data associated with the article are available under the terms of the Creative Commons Zero "No rights reserved" data waiver (CC0 1.0 Public domain dedication).

As noted, non-radioactive DYRK1A assays have been described ^21, 22^. These assays employ similar solution DYRK1A reactions at first stage and then different approaches for measuring the phosphorylated products. One used a phospho-specific antibody to capture products for subsequent immuno/colorimetric detection ^21^, while another used a fluorescein-tagged substrate and analyzed products by high performance liquid chromatography/fluorescence detection ^22^. The above methods have been optimized for sensitivity to measure cellular DYRK1A activity. We do not know whether our ELISA method, at the current stage, affords such level of sensitivity. Nevertheless, as we have demonstrated, our ELISA assay provides sufficient sensitivity for analyzing inhibitor activity with recombinant DYRK1A. Furthermore, because of the standard ELISA protocol, our assay is straightforward to perform with the entire process carried out in a single well. The tools and equipment for adapting this plate-based assay for high throughput automation are widely available, and if necessary, the assay can be refined to further improve the sensitivity. We believe that our assay offers a simple, rapid, and reliable non-radioactive method suitable for replacing the radioactive trace assays in quantifying and screening DYRK1A inhibitors.

## Data availability

The data referenced by this article are under copyright with the following copyright statement: Copyright: © 2017 Liu Y et al.

Data associated with the article are available under the terms of the Creative Commons Zero "No rights reserved" data waiver (CC0 1.0 Public domain dedication). 

**Dataset 1.**

Raw data for Figures 1– 7 and Supplementary Figure are supplied in Excel format.

10.5256/f1000research.10582.d155317 ^42^ Zipped file, containing the following:

**Data for Figure 1. Coating ELISA plate with HT-PRD.** Data (OD _405_) for 0 – 800 ng of coated HT-PRD per well were shown. Background measurements for all Figures (1– 7 and Supplementary Figure) were obtained using wells with PNPP only (no coating, no phosphorylation, and no antibodies), which were performed in parallel with each experimental replicate/triplicate for background correction. The data shown in the files for all Figures have been corrected using averaged background from each set.

**Data for Figure 2. Phosphorylation of coated HT-PRD by DYRK1A.** Data (OD _405_) for 0 – 800 ng coated HT-PRD per well were shown.

**Data for Figure 3. DYRK1A concentration-dependent phosphorylation of coated HT-PRD.** Data (OD _405_) for phosphorylation with 0 – 80 ng HT-497 were shown.

**Data for Figure 4. Time-course phosphorylation of coated HT-PRD by DYRK1A.** Time-course phosphorylation data (OD _405_) with 0–90 min incubation time were shown.

**Data for Figure 5. 3D3 dilution factor determination.** Data (OD _405_) for 3D3 dilution 1:1,000 – 256,000 were shown.

**Data for Figure 6A. Epigallocatechin gallate** ( **EGCG) inhibition profile.** Data (OD _405_) for EGCG 0 – 3.2 μM were shown.

**Data for Figure 6B. Harmine inhibition profile.** Data (OD _405_) for harmine 0 – 3.2 μM were shown.

**Data for Figure 7. ATP competition assay.** Data (OD _405_) for ATP 100 – 800 μM were shown.

**Data for Supplementary Figure. Secondary antibody dilution factor determination.** Data (OD _405_) for secondary antibody dilution 1:1,000 – 256,000 were shown.

## Supplementary materials

**Supplementary Figure. Secondary antibody dilution factor determination.** Phosphorylated HT-PRD in ELISA wells were prepared and probed with 3D3 (1:3000 dilution) followed by serially diluted secondary antibody (from 1000 to 256,000x) similarly as described in Figure 5. Like 3D3 titering, normalized OD _405_ was calculated for plotting (n = 6 for each data point).

.

**Supplementary Table. Estimation of Z’-factor for the ELISA assay performed under standard conditions.**
