# Supplementary material for: An ELISA DYRK1A non-radioactive kinase assay suitable for the characterization of inhibitors
Source: F1000Res. 2017 Mar 24;6:42. Originally published 2017 Jan 13. [Version 2] doi: 10.12688/f1000research.10582.2 (PMC5270589; doi:10.12688/f1000research.10582.2)
Supplement: Supplementary file 3 [file f1000research-6-12095-s0002.tgz › 4c51b7c0-6031-455c-875e-4554c09b09ef.docx]

Supplementary Table. Estimation of Z’-factor for the ELISA assay performed under standard conditions.

|  |  | OD_405_ |  |  |
| --- | --- | --- | --- | --- |
|  | Background | Negative Control | Complete assay | Z’-factor |
| Set 1 | 0.087 | 0.006 | 1.120 | 0.79 |
|  | 0.085 | -0.002 | 1.167 |  |
|  |  | 0.001 | 1.076 |  |
|  |  | 0.008 | 1.265 |  |
|  |  | 0.005 | 1.216 |  |
|  |  |  |  |  |
| Set 2 | 0.092 | -0.002 | 1.142 | 0.82 |
|  | 0.090 | -0.003 | 1.121 |  |
|  |  | 0.000 | 1.113 |  |
|  |  | 0.001 | 1.228 |  |
|  |  | -0.001 | 1.044 |  |
|  |  |  |  |  |
| Set 3 | 0.058 | 0.002 | 0.933 | 0.70 |
|  | 0.055 | 0.006 | 0.910 |  |
|  |  | 0.002 | 0.823 |  |
|  |  | -0.001 | 0.729 |  |
|  |  | 0.006 | 0.857 |  |

Complete assays were performed under standard conditions [200 ng of HT-PRD, 5 ng HT-497, 100 μM ATP, and 30 min kinase reaction at 30°C] and quantified as described in the Methods. Negative controls were assays conducted in parallel but without the addition of HT-497 (DYRK1A). Complete assays and negative controls in each set (5 repeats) were first corrected for the background (PNPP only) and then used for Z’-factor calculation according to Zhang, JH. et al. [1]

1. Zhang JH, Chung TD, Oldenburg KR. A simple statistical parameter for use in evaluation and validation of high throughput screening assays. *J. Biomol. Screen.* 1999; 4(2): 67-73.

PubMed Abstract: <https://www.ncbi.nlm.nih.gov/pubmed/10838414>

Publisher Full Text: <http://journals.sagepub.com/doi/abs/10.1177/108705719900400206>
