# Supplementary material for: An ELISA DYRK1A non-radioactive kinase assay suitable for the characterization of inhibitors
Source: F1000Res. 2017 Mar 24;6:42. Originally published 2017 Jan 13. [Version 2] doi: 10.12688/f1000research.10582.2 (PMC5270589; doi:10.12688/f1000research.10582.2)
Supplement: Peer review history

# Peer review: Referee response for version 1

**DOI:** 10.5256/f1000research.11404.r19700

**Published:** 2017-01-26

**Authors:** Stefan Knapp

**Affiliations:** 1 Nuffield Department of Medicine, Structural Genomics Consortium, University of Oxford, Oxford, UK

I approve the article by Liu et al.

 DYRK1A has developed into an interesting pharmacological target. The assay described in this paper offers an interesting alternative to previously published assays.

 For assay validations the authors made however a poor choice. Epigallocatechin gallate (EGCG) has been identified as a DYRK1 inhibitor but this compound is highly reactive and not a meaningful inhibitor for assay validation (even though there might be clinical benefits using this natural product due to the broad spectrum of published possible cellular activities). Recently the diverse problem using promiscuous, reactive and chemically unstable inhibitors (PAINS) has been highlighted in the literature. Certainly EGCG should not be used as a control compound in any assay.

 However, the data is well presented (antibody source should be described) and a good addition to the kinase assay repertoire that can be used studying DYRK1A inhibitors.

I have read this submission. I believe that I have an appropriate level of expertise to confirm that it is of an acceptable scientific standard.

# Response

**Published:** 2017-03-24

**Authors:** Yu-Wen Hwang

**Affiliations:** NYS Institute for Basic Research, USA

We thank Dr. Knapp for the comments. 

 We agree with Dr. Knapp that EGCG is known to be unstable and is reactive toward many cellular targets. Clearly, EGCG is not a suitable reagent for validating biological assays. However, in a defined *in vitro* system consisting of only DYRK1A, substrate, ATP, and buffer, like our ELISA-based assay, EGCG is a well-behaved small molecule. Its property is predictable and can be reliably measured under such conditions as has been done by us and others. In addition, EGCG inhibits DYRK1A through a non-ATP-competitive mechanism. Pairing EGCG with ATP-competitive inhibitors such as harmine provides different prospects for assessing the ELISA assay.   

 The antibody used in the study, 3D3, has been deposited with the Developmental Studies Hybridoma Bank of the University of Iowa.

# Peer review: Referee response for version 1

**DOI:** 10.5256/f1000research.11404.r19325

**Published:** 2017-01-18

**Authors:** Walter Becker

**Affiliations:** 1 Institute of Pharmacology and Toxicology, Aachen University, Aachen, Germany

Liu and coworkers have developed an ELISA-based assay for the protein kinase DYRK1A and show that their assay compares well with the traditional radioactive assays in the analysis of DYRK1A inhibitors. As correctly pointed out the authors, a previous ELISA-based DYRK1A assay developed in my own lab had been optimized for sensitive detection of endogenous DYRK kinases, while the new ELISA assay is more straightforward and provides a simple and rapid method for analyzing inhibitor activity with recombinant DYRK1A. The inhibitions curves with the DYRK1A inhibitors EGCG and harmine support the conclusion “that the ELISA platform demonstrated here is a viable alternative to the traditional radioactive tracer assays for analyzing DYRK1A inhibitors”.

 This methods article clearly represents a substantial modification of an existing procedure. The study is well designed, the methods and the analysis of the results are appropriately described and the conclusions are justified on the basis of the results. In summary, this is sound and useful study that will be valuable for other researchers in the field.

**Recommendations**

- In the 2 ^nd^ paragraph the introduction, presenilin is misspelled.
- I appreciate the identification of the used antibodies in the method section by their accession numbers in the antibody registry. Direct links to this registry would be helpful for the reader.
- For the convenience of the reader, I suggest to include the harmine inhibition curve (supplemental Fig. 2) in the main text as a second panel in Fig. 6.
- The final statement of the manuscript suggests the use their assay for the screening of DYRK1A inhibitors. It may be worth to validate this application by determining the Z’–factor of the assay (according to Zhang et al. 1999 ^1^).
- The availability of plasmids and antibodies should be indicated. Are the plasmids available from the authors or from Addgene? Will the authors make the hybridoma clone for the pSer857 3D3 antibody available or can the antibody be commercially purchased?
- It must be stated in the figure legends whether the error bars show SD or SEM. I suggest to show SD, which provides the reader a measure for the experimental error.
- The raw data for the figures are provided in the KaleidaGraph format and were not accessible to me. Is it possible to submit as Excel or PDF?
- The authors may consider to include the term "kinase" in the title to enhance visibility of theit article to readers not aware of DYRK1A.

I have read this submission. I believe that I have an appropriate level of expertise to confirm that it is of an acceptable scientific standard.

## References

[1] ZhangJHChungTDOldenburgKR: A Simple Statistical Parameter for Use in Evaluation and Validation of High Throughput Screening Assays. *J Biomol Screen*.1999;4(2) :67-73 1083841410.1177/108705719900400206

# Response

**Published:** 2017-03-24

**Authors:** Yu-Wen Hwang

**Affiliations:** NYS Institute for Basic Research, USA

We thank Dr. Becker for comments and suggestions. We have revised the article and addressed most of the questions. Here is the summary of changes.

- The typo is corrected.
- Direct link to antibody registry is added.
- Supplementary Figure 2 for harmine inhibition is now incorporated in the main text as Figure 6B.
- The Z’-factor for the ELISA assay has been estimated. The results are shown in the Supplementary Table. 
- We have deposited the vectors (pHT-497 and pHT-PRD) and antibody 3D3 with Addgene and the Developmental Studies Hybridoma Bank of the University of Iowa, respectively.  
- The error bars are SEM. This is now indicated in the revised article. 
- The raw data in Excel format have been submitted.
- The term “kinase” is added to the title.
